# Supplementary material for: A Similar Lifetime CV Risk and a Similar Cardiometabolic Profile in the Moderate and High Cardiovascular Risk Populations: A Population-Based Study
Source: J Clin Med. 2021 Apr 9;10(8):1584. doi: 10.3390/jcm10081584 (PMC8069041; doi:10.3390/jcm10081584)
Supplement: Supplementary file 1 [file jcm-10-01584-s001.pdf]

Table S1. Results of SWLS, EQ-VAS, BDI analysis in general population

Variable		SWLS	P	EQ-VAS	P	BDI	P
History of hypertension	no	23.4±5.3	0.019	79.1±13.7	<0.001	6.5±6.3	<0.001
	yes	22.4±5.2		70.8±15.9		8.4±7.1	
History of hypercholesterolemia	no	23.4±5.2	0.041	77.9±14.9	<0.001	6.6±6.5	<0.001
	yes	22.5±5.5		73.8±14.4		8.0±6.7	
History of diabetes	no	23.1±5.3	0.215	77.4±14.5	<0.001	6.8±6.5	<0.001
	yes	22.4±4.8		66.9±15.9		10.21±7.2	
BP ≥140 and/or ≥90mmHg	no	23.1±5.4	0.674	77.1±14.8	0.047	7.1±6.5	0.484
	yes	22.9±5.0		75.3±14.9		6.9±6.8	
Hypercholesterolemia*	no	23.2±5.4	0.717	80.1±13.5	<0.001	7.0±6.3	0.924
	yes	23.0±5.3		75.3±15.1		7.0±6.7	
Hyperglycemia**	no	23.2±5.3	0.939	78.0±14.3	<0.001	6.7±6.5	0.002
	yes	23.2±5.2		71.4±13.9		8.6±6.5	

BDI: Beck Depression Inventory; BP: blood pressure; CV: cardiovascular; EQ-VA: Euro Quality of Life Visual Analogue Scale; LDL-C: low-density cholesterol; OGTT: oral glucose tolerance test; SWLS: Satisfaction With Life Scale

*Total cholesterol >190mg% or LDL-C >116mg% in low CV class, >100mg% in moderate CV class, >70mg% in high CV class, >55mg% in very-high CV class;

** Fasting glucose ≥ 126 mg/dl or OGTT 120min. ≥ 200mg/dl
